# Healthcare-Associated Bloodstream Infections Due to Multidrug-Resistant *Acinetobacter baumannii* in COVID-19 Intensive Care Unit: A Single-Center Retrospective Study

**DOI:** 10.3390/microorganisms11030774

**Published:** 2023-03-17

**Authors:** Karolina Dobrović, Tea Škrobo, Katarina Selec, Marko Jelić, Rok Čivljak, Jasminka Peršec, Sanja Sakan, Nikolina Bušić, Antonija Mihelčić, Sonja Hleb, Arjana Tambić Andrašević

**Affiliations:** 1 Department of Clinical Microbiology and Hospital Infections, Dubrava University Hospital, Av. Gojka Šuška 6, 10000 Zagreb, Croatia; kdobrov@kbd.hr (K.D.);; 2 Department for Clinical Microbiology, University Hospital for Infectious Diseases “Dr. Fran Mihaljević”, Mirogojska 8, 10000 Zagreb, Croatia; mjelic@bfm.hr (M.J.); atambic@bfm.hr (A.T.A.); 3 Department for Acute Respiratory Tract Infections, University Hospital for Infectious Diseases “Dr. Fran Mihaljević”, Mirogojska 8, 10000 Zagreb, Croatia; 4 Department for Infectious Diseases, School of Medicine, University of Zagreb, Šalata 3, 10000 Zagreb, Croatia; 5 Department of Anesthesiology, Reanimatology and Intensive Care Medicine, Dubrava University Hospital, Av. Gojka Šuška 6, 10000 Zagreb, Croatia; 6 School of Dental Medicine, University of Zagreb, Gundulićeva 5, 10000 Zagreb, Croatia; 7 Department of Internal Medicine, Dubrava University Hospital, Av. Gojka Šuška 6, 10000 Zagreb, Croatia

**Keywords:** *Acinetobacter baumannii*, *bla*_OXA-23_, bloodstream infection, carbapenemase genes, carbapenem resistance, COVID-19, healthcare-associated infections, intensive care units, multidrug resistance

## Abstract

Healthcare-associated infections are an emerging cause of morbidity and mortality in COVID-19 intensive care units (ICUs) worldwide, especially those caused by multidrug-resistant (MDR) pathogens. The objectives of this study were to assess the incidence of bloodstream infections (BSIs) among critically ill COVID-19 patients and to analyze the characteristics of healthcare-associated BSIs due to MDR *Acinetobacter baumannii* in an COVID-19 ICU. A single-center retrospective study was conducted at a tertiary hospital during a 5-month period. The detection of carbapenemase genes was performed by PCR and genetic relatedness by pulsed-field gel electrophoresis (PFGE) and multilocus-sequence typing. A total of 193 episodes were registered in 176 COVID-19 ICU patients, with an incidence of 25/1000 patient-days at risk. *A. baumannii* was the most common etiological agent (40.3%), with a resistance to carbapenems of 100%. The *bla*_OXA-23_ gene was detected in ST2 isolates while the *bla*_OXA-24_ was ST636-specific. PFGE revealed a homogeneous genetic background of the isolates. The clonal spread of OXA-23-positive *A. baumannii* is responsible for the high prevalence of MDR *A. baumannii* BSIs in our COVID-19 ICU. Further surveillance of resistance trends and mechanisms is needed along with changes in behavior to improve the implementation of infection control and the rational use of antibiotics.

## 1. Introduction

For almost three years now, the coronavirus disease 2019 (COVID-19) pandemic caused by severe acute respiratory syndrome coronavirus 2 (SARS-CoV-2) has been a leading health problem in countries worldwide. By the beginning of 2023, more than 750 million cases of COVID-19 and over 6.5 million deaths had been reported worldwide [1]. Although most people infected with SARS-CoV-2 develop a mild to moderate disease, some progress to life-threatening pneumonia that warrants hospitalization with up to approximately 16% requiring admission to an intensive care unit (ICU) [2,3]. It is known that ICU patients are susceptible to healthcare-associated infections (HAIs), especially ICU-acquired bloodstream infections (BSIs) [4]. Recent research shows that the risk of ICU-acquired BSIs is higher for COVID-19 than non-COVID-19 critically ill patients after seven days of ICU stay [5].

On the other hand, healthcare-associated BSIs caused by multidrug-resistant (MDR) *Acinetobacter baumannii* represent an emerging problem among ICU patients because of their association with high morbidity and mortality, limited therapeutic options, and high treatment costs [6,7]. Apart from having the propensity to rapidly develop antimicrobial resistance, this organism has the ability to successfully survive, persist, and be transmitted in ICU settings, mostly due to the contamination of respiratory equipment and transmission via healthcare workers’ hands [8]. Carbapenems used to be the cornerstone for the treatment of severe HAIs caused by *A. baumannii* [9]. However, Croatia is a country with a high prevalence of carbapenem-resistant *A. baumannii* strains, with the carbapenem resistance rate exceeding 90% [10]. Carbapenem-resistant *A. baumannii* strains mostly exhibit an MDR phenotype and are also resistant to gentamicin, amikacin, ciprofloxacin, and trimethoprim–sulfamethoxazole.

The resistance of *A. baumannii* to antibiotics is often a combination of several mechanisms, including porin loss, the overexpression of the efflux pump, and the production of several types of beta-lactamases [11]. Carbapenem resistance in *A. baumannii* is mostly due to the production of carbapenem-hydrolyzing enzymes, Ambler class D oxacillinases (OXAs) among which are intrinsic *bla*_OXA-51_ carbapenemase and several enzymes coded by acquired *bla*_OXA_ genes (*bla*_OXA-23_, *bla*_OXA-40_, and *bla*_OXA-58_). Class B metallo-β-lactamases (MBLs) (*bla*_VIM_, *bla*_IMP_, *bla*_NDM_) are less frequently found [11,12]. Although oxacillinases weakly hydrolyze carbapenems, the overexpression of the *bla*_OXA_ genes and higher resistance can be promoted by the insertion of mobile elements, such as IS*Aba1* [13]. Until now, there have been numerous reports worldwide of the dissemination of carbapenem-resistant *A. baumannii* strains possessing either OXA or MBL genes, among which molecular typing has revealed three successful clones, designated as international clones (ICs) I–III [12,14,15,16].

Given the increasing number of bloodstream infections observed among the patients admitted to our COVID-19 ICU, the objectives of this study were to assess the incidence of healthcare-associated BSIs among critically ill COVID-19 patients and to analyze the clinical and microbiological characteristics of healthcare-associated BSIs due to MDR *A. baumannii* in COVID-19 intensive care unit. Furthermore, we describe the hospital environment and infection control practices adopted during the studied period.

## 2. Materials and Methods

This was a single-center retrospective study conducted at the Dubrava University Hospital, a tertiary care teaching hospital in Zagreb, Croatia, during the second wave of the COVID-19 pandemic, from 1 October 2020 to 28 February 2021. All ICU patients treated for severe COVID-19 with healthcare-associated BSIs were included in the study. The ICU environment, infection control practices, the use of personal protective equipment, and the contact isolation of patients colonized or infected with MDR microorganisms were all observed.

### 2.1. Setting

The Dubrava University Hospital is one of the largest hospitals in the Republic of Croatia, with a capacity of 650 patient beds. More than 25,000 patients are treated in the inpatient departments annually, while more than 1,500,000 health services are provided to more than 400,000 patients in the outpatient department. In March 2020, the hospital was converted into a primary respiratory intensive care center (PRIC) for the treatment of patients with severe COVID-19. Prior to the COVID-19 pandemic, the hospital had an ICU capacity of 35 beds in three ICU subunits. In a short period of time, that number gradually increased to 85 ICU beds distributed in six ICU subunits. Due to organizational changes, the microbiology laboratory was closed and microbiological diagnosis was performed at another hospital.

### 2.2. Definitions and Data Collection

COVID-19 diagnosis was defined by the typical clinical presentation in the presence of a positive real-time polymerase chain reaction (RT-PCR) for SARS-CoV-2 on a nasopharyngeal swab. An ICU-acquired BSI was defined by at least one positive blood culture for bacterial or fungal microorganisms in a patient with systemic signs of infection, diagnosed >48 h after ICU admission. The isolation of the same microorganism from the bloodstream within 14 days was not considered a novel episode [17]. For coagulase-negative staphylococci (CoNS) and other common skin contaminants, at least two sets of blood cultures positive for the same pathogen were necessary to define a clinically significant BSI [18]. A polymicrobial bloodstream infection was defined by the isolation of two or more microorganisms from a single blood culture set, or from different blood culture sets obtained within 48 h [19,20].

Patients’ data were retrospectively extracted from computerized hospital databases. The following information was reviewed: demographic parameters (age in years and gender), comorbid conditions (cardiomyopathy, peripheral artery disease, chronic obstructive pulmonary disease (COPD), chronic renal insufficiency, diabetes mellitus, and neurologic disease), COVID-19 characteristics at ICU admission (the duration of the disease prior to admission, the presence of bilateral pneumonia, PaO_2_/FiO_2_) and outcome, including fatal outcome in the ICU. The severity of the clinical condition was determined using Sequential Organ Failure Assessment (SOFA), Acute Physiology and Chronic Health Evaluation II (APACHE II), and Simplified Acute Physiology Score II (SAPS II) scores calculated at the time of ICU admission. Furthermore, we analyzed the immunomodulatory and antimicrobial therapy that patients received for COVID-19 treatment, the duration of antimicrobial therapy prior to BSI, as well as the targeted therapy for *A. baumannii* BSI.

### 2.3. Laboratory Procedures and Statistical Analysis

In general, two sets of blood cultures were taken whenever clinically indicated. In patients with central venous catheters, one set was drawn from a central venous catheter and one from a peripheral venipuncture site. A blood culture set consisted of one aerobic and one anaerobic bottle. Blood cultures were collected aseptically and then inoculated in an automated BacT/Alert system (bioMérieux, Marcy l’Étoile, France). Blood cultures were continuously processed on a 24 h per day, 7 days per week schedule and a preliminary report based on a blood culture Gram-stain was reported to the responsible clinician on a “24/7” basis. Identification of blood culture isolates was based on routine laboratory techniques, which included Gram-staining, biochemical tests, and an automated VITEK-2 system. Antimicrobial susceptibility testing was performed by a disk diffusion test for ampicillin–sulbactam, imipenem, meropenem, gentamicin, amikacin, ciprofloxacin, and trimethoprim–sulfamethoxazole, and by the microbroth dilution method for the detection of the minimal inhibitory concentration (MIC) for colistin (Mikrolatest^®^ MIC Colistin, Erba Lachema s.r.o., Brno, Czech Republic) and for confirmation, if necessary, an additional test was performed (MIC-Strip Colistin, Bruker Daltonics GmbH & Co., KG Bremen, Germany). The interpretation of antimicrobial susceptibility was performed according to the European Committee on Antimicrobial Susceptibility Testing (EUCAST), except for ampicillin–sulbactam and *A. baumannii*, in which case the interpretation according to the Clinical Laboratory Standards Institute (CLSI) was applied [21,22]. Susceptibility testing for cefiderocol was not performed since it was not available in Croatia during the studied period.

### 2.4. Molecular Analysis of Carbapenem Resistance Determinants

PCR was used for the detection of class D carbapenemase genes (*bla*_OXA-23_, *bla*_OXA-24_, *bla*_OXA-51_, *bla*_OXA-58_) [23] and class B metallo-β-lactamases (*bla*_VIM_, *bla*_IMP_, *bla*_NDM_) [24]. The presence and localization of IS*Aba(1–3)* elements upstream of the *bla*_OXA_ genes were assessed by PCR using a combination of primers specific for the insertion sequence IS*Aba(1–3)* and for class D carbapenemase genes (*bla*_OXA-23_, *bla*_OXA-24_, *bla*_OXA-51_, *bla*_OXA-58_), as previously described [25]. All PCR reactions were performed on Veriti thermal cycler (Thermo Fisher Scientific, Waltham, MA, USA).

### 2.5. Molecular Epidemiology

Pulsed-field gel electrophoresis (PFGE) of *ApaI* digested genomic DNA was used in order to assess the genetic relatedness of all the *A. baumannii* isolates included in this study. PFGE was performed using the CHEF-III System (Bio-Rad Laboratories, Hercules, CA, USA) according to the previously described protocol [26]. PFGE band pattern similarity calculation and clustering were performed using the dice coefficient (1.5% tolerance) and unweighted pair group method with arithmetic mean (BioNumerics v7.6, Sint-Martens-Latem, Belgium). The threshold of ≥85% similarity was used for the genetic cluster assignment. Multilocus sequence typing (MLST) was performed on representative isolates according to the “Pasteur scheme” protocol described on the *A. baumannii* MLST web site (https://pubmlst.org/primers-used-mlst-acinetobacter-baumannii-complex-pasteur-scheme; accessed on 1 March 2021) [27]. All the isolates were assigned to International Clone Lineages (IC) using the sequence-based typing (SBT) of *bla*_OXA-51_ [28].

### 2.6. Statistical Analysis

The incidence rate of ICU-acquired BSIs in the study population was calculated as the number of BSI episodes per 1000 patient-days at risk (defined as the cumulative days of stay elapsed from 48 h after ICU admission to death or discharge from the ICU). The demographic and clinical data are recorded as absolute numbers, percentages, or median values—all calculated using Microsoft Excel (2016, Redmond, WA, USA).

## 3. Results

During the study period, a total of 193 episodes of ICU-acquired BSIs were registered in 176 patients, with an incidence rate of 25 episodes per 1000 patient-days at risk. A significant proportion (73/193, 37%) of the ICU-acquired BSIs were polymicrobial.

### 3.1. Bacterial Isolates and Resistance

Two hundred and seventy-eight isolates were identified. The majority (160/278, 57.6%) were Gram-negative bacteria, with *A. baumannii* being the most prevalent pathogen (112/278, 40.3%), followed by *Klebsiella pneumoniae* (24/278, 8.6%) and *Pseudomonas aeruginosa* (6/278, 2.2%). All the *A. baumannii* isolates were resistant to carbapenems. Among the *K. pneumoniae* isolates, 21/24 (87.5%) were extended spectrum beta lactamase (ESBL) and OXA-48 carbapenemase producers. Other Gram-negative bacteria were *Escherichia coli*, *Enterobacter cloacae*, *Serratia marcescens*, *Providencia stuartii*, *Proteus mirabilis*, *Stenotrophomonas maltophilia*, *Klebsiella oxytoca*, *Klebsiella aerogenes*, and *Moraxella* species, each in a percentage of less than 2%.

Among the Gram-positive bacteria, *Enterococcus* spp. was the most common species (44/278, 15.8%), and included 19 *Enterococcus faecalis* and 25 *Enterococcus faecium* isolates. Among all of *Enterococcus* spp., 9 (20.5%) isolates were vancomycin-resistant *Enterococcus* (VRE). The next most frequent Gram-positive isolates following enterococci were coagulase-negative staphylococci (37/278, 13.3%) and *Staphylococcus aureus* (24/278, 8.6%). Among the *S. aureus* isolates, 20/24 (83%) were methicillin-resistant *S. aureus* (MRSA). Other Gram-positive bacteria that were isolated were *Corynebacterium* species, *Listeria monocytogenes* and group G beta-hemolytic streptococci, each in a percentage of less than 2%. *Candida* species accounted for 1% of all isolates.

**Figure 1:**
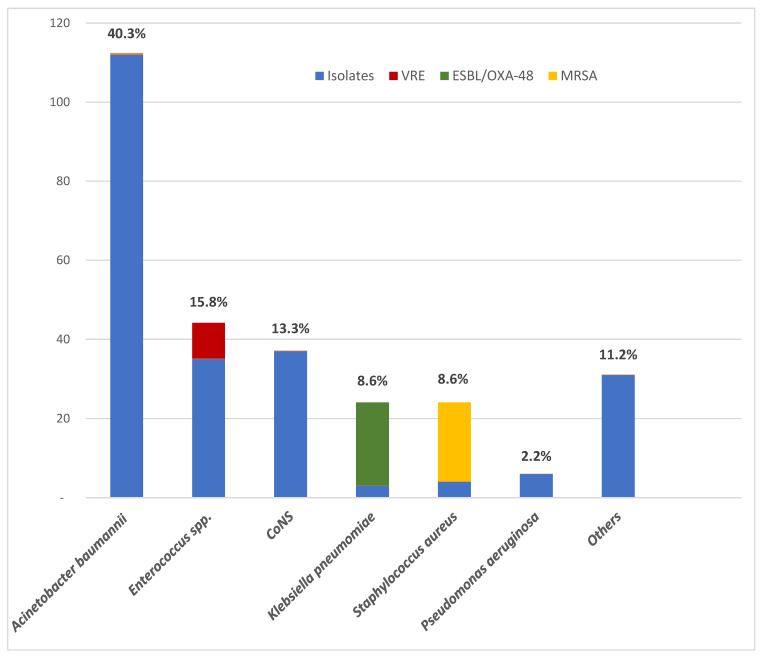
The most common etiological agents of healthcare-associated bloodstream infections in the COVID-19 intensive care unit at the Dubrava University Hospital during the second wave of the COVID-19 pandemic (1 October 2020–28 February 2021) (N = 278). Legend: CoNS—coagulase-negative staphylococci; ESBL—extended-spectrum beta-lactamase; MRSA—methicillin-resistant *Staphylococcus aureus*; OXA-48—oxacillinase (OXA)–48; VRE—vancomycin-resistant enterococci.

The most common etiological agents of healthcare-associated BSIs in 176 COVID-19 patients (193 episodes) in the COVID-19 intensive care unit at the Dubrava University Hospital during the second wave of COVID-19 pandemic are shown in Figure 1.

### 3.2. Patients with BSI Caused by MDR A. baumannii

#### 3.2.1. Characteristics of Patients

Out of a total of 176 patients, 106 (60.2%) were infected with MDR *A. baumannii.* The characteristics of the 106 patients with healthcare-associated bloodstream infections due to MDR *A. baumannii* in COVID-19 ICU are summarized in Table 1.

The median age of the patients was 72 (range 89–48) years, and 83 (78.3%) were male. The majority of the patients (78/106, 73.6%) had at least one comorbidity. Among them, a significant prevalence of diabetes mellitus (40/106, 37.7%) was found followed by peripheral artery disease (22/106, 20.7%), coronary artery disease (19/106, 17.9%), cardiomyopathy (12/106, 11.3%), neurologic disease (11/106, 10.4%), chronic renal insufficiency (9/106, 8.5%), and chronic obstructive pulmonary disease (6/106, 5.7%). The median duration of the disease prior to admission to the ICU was 10 (range 1–36) days. Bilateral pneumonia was confirmed by the chest radiography in 99 (93.4%) of the patients.

The median SOFA score was 4, the APACHE II score was 13, and the SAPS II score was 30.5. At the ICU admission, 99 (93.4%) of the patients had bilateral pneumonia with a median PaO_2_/FiO_2_ of 67.9.

Regarding the treatment of severe COVID-19, 65 (61.3%) of the patients received immunomodulatory therapy, mainly dexamethasone or methylprednisolone, while 21 (19.8%) patients received antivirals, mainly remdesivir.

A significant number (99/106, 93.4%) of the patients had antibiotic exposure in the 30 days preceding a positive blood culture. They received at least one dose of the following agents: ceftriaxone, meropenem, piperacillin/tazobactam, imipenem, vancomycin, colistin, azithromycin, cefepime, linezolid, amoxicillin/clavulanic acid, ampicillin/sulbactam, clindamycin, amikacin, gentamicin, intravenous Fosfomycin, or metronidazole. The most prescribed antibiotics were ceftriaxone (in 57/106, 53.8%), meropenem (in 24/106, 22.6%), piperacillin/tazobactam (in 18/106, 16.9%), and amoxicillin/clavulanic acid (in 12/106, 11.3%). Sixty (56.6%) patients had received more than one antibiotic prior to a BSI. Subsequently, antibiotics were changed based on the blood culture report and clinical improvement. For the targeted treatment of a BSI, 47 (44.3%) of the patients received colistin as monotherapy, while in 29 (27.3%) of the patients, colistin was combined with another antibiotic, depending on the result of the antimicrobial susceptibility testing. The antibiotics most frequently combined with colistin were meropenem, ampicillin/sulbactam, and linezolid. Furthermore, nine patients received more than one antibiotic in addition to colistin. Only six (5.7%) patients received therapy that did not include colistin: they were treated with ampicillin/sulbactam in monotherapy or in combination with linezolid for presumed polymicrobial infection.

Only 15 (14.2%) of the patients were discharged from the hospital while 91 (85.8%) had a fatal outcome during their treatment in the ICU.

#### 3.2.2. Antimicrobial Resistance Pattern

The antimicrobial resistance pattern of the MDR *A. baumannii* isolates is shown in Table 2.

All the *A. baumannii* isolates were resistant to carbapenems (imipenem and meropenem) and to ciprofloxacin. Resistance to gentamicin was registered in 111 (99.1%) of the patients, to amikacin in 100 (89.3%), and to trimethoprim–sulfamethoxazole in 43 (38.4%). There was a low incidence of resistance to ampicillin/sulbactam (11/112, 9.8%) and colistin (4/105, 3.8%).

#### 3.2.3. Carbapenem Resistance Determinants

The oxacillinase gene *bla*_OXA-51_ was detected in all the isolates included in this study. The detection of *bla*_OXA-23_ and *bla*_OXA-24_ was sequence-type-specific, resulting in *bla*_OXA-23_ gene detection in all ST2 isolates (83%), while *bla*_OXA-24_ was detected only in ST636 (17%). The presence of class B metallo-β-lactamases was not detected in any of the *A. baumannii* isolates included in this study. The IS*Aba1* genetic element was detected in all the ST2 isolates upstream of the *bla*_OXA-23_ gene.

#### 3.2.4. Molecular Epidemiology Results

PFGE analysis revealed the homogeneous genetic background of the isolate. Isolates were delineated according to the three main genetic clusters (A–C) (≥85% similarity) and eight singleton PFGE profiles. Most of the isolates analyzed belonged to cluster A (*n* = 45, 63%), followed by cluster B (*n* = 12, 17%), and cluster C (*n* = 7, 10%), respectively. Genetic clusters A and C corresponded to ST2, while cluster B corresponded to ST636 by MLST. All isolates were designated as international clone 2 (IC2).

### 3.3. Hospital Environment and Infection Control

We encountered several problems that the hospital was facing at the time. In a short period, due to the COVID-19 pandemic and the need for additional ICU beds, the number of ICU beds increased from 35 in three ICU subunits to 85 in six ICU subunits (pop-up ICUs). An additional problem was the need to transfer patients with pre-existing MDR infections from other institutions (e.g., surgical, neurological, internal medicine, and hematological ICUs) to our hospital due to COVID-19. ICU beds were placed too close to each other, and the environment was insufficiently cleaned and disinfected at that time. The overcrowding of the ICUs made the isolation of patients with MDR pathogens very difficult. Due to the labor shortage and overload of work in the ICUs, healthcare workers were transferred from other hospitals and departments to our ICUs. The correct use of personal protective equipment has been a particular challenge. Hand hygiene was inadequate; there was the widespread use of double or triple gloving and the decontamination of gloves with alcohol.

## 4. Discussion

In the present study, we found an incidence rate of 25/1000 patient-days at risk for healthcare-associated BSIs in COVID-19 ICU, with a high prevalence of resistant Gram-negative microorganisms as causative agents, particularly MDR *A. baumannii* (40.3%). In addition to the high incidence of *A. baumannii*, the other prevalent Gram-negatives were ESBL and OXA-48 carbapenemase producing *K. pneumoniae*, while high percentages of MRSA and VRE were observed among the Gram-positives. In comparison to the data found in this study, during the same period prior to the COVID-19 pandemic (from 1 October 2019 to 28 February 2020), the incidence rate of healthcare-associated BSIs was much lower (5/1000 patient-days at risk) while neither OXA-48 producing *Enterobacteriales* nor MRSA and VRE isolates were confirmed among healthcare-associated BSIs in our hospital (unpublished work).

Similar observations were described in previous reports, with a prevalence of Gram-negative microorganisms of up to 82.8% [29,30,31,32]. Conversely, in a study by Giacobbe et al. [33] and a few other studies [20,34], an increased prevalence of Gram-positive microorganisms with a predominance of *Enterococcus* spp. and *S. aureus* was reported. This disparity in prevalence may be a consequence of different patient settings, duration of hospital stays, or local diversity in endemically present pathogens as well as local antimicrobial policies. Furthermore, in the aforementioned studies, data were collected from COVID-19 wards within a multipurpose hospital, while the Dubrava Clinical Hospital was exclusively a COVID-19 center during the study period.

A possible explanation for the unexpectedly high frequency of BSIs due to MDR *A. baumannii* could be the excessive usage of broad-spectrum antimicrobial agents as initial empirical therapy in patients with severe COVID-19. In our study, 93.4% of the patients received initial empirical antibiotic therapy prior to the BSI episode. Furthermore, 56.6% of our patients received a combination of two or more antibiotics upon admission to the ICU.

In a recent systematic review by Chedid et al., the rate of antibiotic use among COVID-19 hospitalized patients was 74%, mainly in an empirical setting. However, this study also included patients with mild and moderate COVID-19, not only ICU patients, as in our study [35]. He et al. reported a significant positive association between the use of a combination of two or more antibiotics in cases of COVID-19 and nosocomial infection [36]. It is possible that the excessive usage of antibiotics contributed to the outbreak of MDR *A. baumannii* BSIs in our COVID-19 ICUs. Moreover, several recent studies did not find a significant difference regarding antibiotic use among surviving and non-surviving COVID-19 patients [37,38,39].

Another additional factor contributing to the high incidence of healthcare-associated BSIs due to MDR *A. baumannii* could be the endemical presence of MDR *A. baumannii* strains in Croatian hospitals. According to the Croatian Committee for Antibiotic Resistance Surveillance of the Croatian Academy of Medical Science, the rate of carbapenem resistance in *A. baumannii* isolates exceeds 90% in many Croatian hospitals. Resistance to carbapenems in *A. baumannii* has rapidly increased from less than 10% in 2008 to more than 60% in 2011, reaching 90% in 2020 in Croatia [10]. Similar situations were observed in other neighboring countries, such as Italy, Hungary, Serbia, Romania, Bulgaria, and Greece where carbapenem resistance exceeds 50% [15,40,41].

Regarding the antimicrobial treatment of MDR *A. baumannii* infections, successful options are limited due to the high rates of resistance to first-line antibiotics, such as beta-lactam antibiotics, carbapenems, and fluoroquinolones. Therefore, second-line agents, including polymyxins (colistin) and tetracycline derivatives (minocycline and tigecycline), are recommended [42,43]. Furthermore, a combination of ampicillin–sulbactam was also found to be effective against severe infections caused by MDR *A. baumannii* [44]. Even a colistin–glycopeptide combination has been shown to be synergistic against MDR Gram-negatives, especially *A. baumannii*, and was shown to be a protective factor for mortality if administered for ≥5 days [45]. All our patients received either colistin or ampicillin–sulbactam alone or together immediately after a clinical microbiologist reported *A. baumannii* as a causative agent of BSI.

Nevertheless, the findings of our study revealed a disturbingly high mortality rate (85.8%) among COVID-19 patients with MDR *A. baumannii* BSI. A study conducted in China on a similar group of patients, although without COVID-19, reported a mortality rate of 69.4% [46]. In addition, other studies have reported 21–50% mortality in COVID-19 patients with associated BSI [20,33,34]. On the other hand, in a recent study by Russo et al., the 30-day mortality among COVID-19 ICU patients with associated MDR *A. baumanni* infection was 81% [6]. Falagas et al. also found infection or colonization with MDR *A. baumannii* to be associated with higher mortality [47]. The data above are in concordance with our findings, suggesting that clinicians should be particularly alert in the case of BSI caused by *A. baumannii* during the course of COVID-19 pneumonia, which is associated with high mortality.

In our study, we found that resistance to carbapenems is based on the production of carbapenem-hydrolyzing class D oxacillinases: *bla*_OXA-23_ and *bla*_OXA-24_. Furthermore, an IS*Aba1* genetic element was detected in all the ST2 isolates upstream of *bla*_OXA-23_. These mechanisms of resistance were previously documented worldwide [14,15,16] and seem to be the most prevalent in our BSI isolates. In Croatia, carbapenem resistance in *A. baumannii* associated with the upregulation of the *bla*_OXA-51_ gene by IS*Aba1* was previously reported [48].

In the present study, the MLST results correlated with PFGE clustering. The homogenous genetic background of the analyzed isolate collection was dominated by a single clone, indicating that the outbreaks were mediated through the spread of the ST2 CRAB lineage during the study timeline in our hospital. Various studies have reported the worldwide distribution of *A. baumannii* ST2/ICII with the *bla*_OXA-23_ gene [14,49], while ST636 *bla*_OXA24_ was less frequently reported [15,50]. MDR IC2 has been a well-established endemic clone in Croatia since 2009 [51]. The first clinical isolates of *A. baumannii* with reduced sensitivity to carbapenems were registered from 2002 to 2007. A study presenting the genotyping results of those isolates demonstrated the presence of the European clone 1 (later IC1) [48]. In 2009, there was an epidemic spread of a new clone belonging to IC2, which is the predominant clone in most Croatian hospitals today [16,52,53].

The abrupt increase in the numbers of critically ill COVID-19 patients admitted to our ICU during the study period led to the neglect of usual infection control practices that had been previously well established. Focus on protecting healthcare workers from the new and relatively unknown COVID-19 has sometimes obscured the importance of personal protective equipment as part of basic infection control measures. The data obtained from the literature also suggest that the COVID-19 pandemic was associated with a less effective implementation of infection control measures among healthcare workers [54,55]. At the beginning of the pandemic, healthcare workers were mainly focused on self-protection rather than the prevention of the cross-transmission of MDR pathogens among patients [55]. Another study conducted in Hong Kong during an outbreak of severe acute respiratory syndrome (SARS) observed an increase in MRSA acquisition in the ICU, suggesting that the excessive usage of gloves may have contributed to reduced compliance with hand hygiene among healthcare workers [56]. It is, therefore, possible that the inadequate implementation of infection control measures played a significant role in the episodes of cross-contamination that eventually increased the number of BSIs in our ICU during the COVID-19 pandemic.

Our study has several limitations. It is a single-center study conducted in a setting with a high prevalence of MDR pathogens, thus limiting the generalizability of our results. Other mechanisms of resistance, apart from carbapenemases, such as porin loss or the overexpression of efflux pumps that could contribute to carbapenem resistance, were not analyzed in this study. The lack of comparative data makes it difficult to ascertain the extent to which organizational changes contributed to the increase in the frequency of BSIs in our COVID-19 ICU.

## 5. Conclusions

The results of our study suggest that the clonal spread of OXA-23 positive *A. baumannii* was responsible for the high prevalence of MDR *A. baumannii* BSIs in the antibiotic-rich COVID-19 ICU environment in our hospital. Further investigations are needed to determine whether the early prescribing of antibiotics in cases of severe COVID-19 is justified, with emphasis on the potential spread of MDR pathogens and secondary healthcare-associated BSIs. In the future, surveillance of resistance trends and mechanisms is needed to streamline research and innovations in the development of new antimicrobials. From our perspective, effective class D beta-lactamase inhibitors are urgently needed to combat severe MDR *A. baumannii* infections. Moreover, a major focus should be on education, as well as behavioral changes in the area of antimicrobial stewardship and infection prevention and control.

## Figures and Tables

**Table 1:** The characteristics of patients with healthcare-associated bloodstream infections due to multidrug-resistant *Acinetobacter baumannii* in the COVID-19 intensive care units at the Dubrava University Hospital during the second wave of the COVID-19 pandemic (1 October 2020–28 February 2021) (N = 106).

Variable	N (%)
Age, median (range), in years	72 (89–48)
Male (%)	83 (78.3)
Comorbidity *	
Without comorbidity	28 (26.4)
With comorbidity	78 (73.6)
Diabetes mellitus	40 (37.7)
Peripheral artery disease	22 (20.7)
Coronary artery disease	19 (17.9)
Cardiomyopathy	12 (11.3)
Neurologic disease	11 (10.4)
Chronic renal insufficiency	9 (8.5)
COPD	6 (5.7)
**Characteristics at ICU admission**	
Duration of disease prior to admission, mean (range), in days	10 (1–36)
Bilateral pneumonia	99 (93.4)
PaO_2_/FiO_2_ (median)	67.9
SOFA score (median)	4
APACHE II score (median)	13
SAPS II score (median)	30.5
**Treatment and outcome**	
Antiviral therapy	21 (19.8)
Immunomodulatory therapy	65 (61.3)
ATB treatment prior to BSI:	99 (93.4)
Ceftriaxone	57 (53.8)
Meropenem	24 (22.6)
Piperacillin/tazobactam	18 (16.9)
Amoxicillin/clavulanic acid	12 (11.3)
Duration of ATB treatment for BSI (median days, mean)	6 (8.3)
**ATB treatment of BSI**	
Colistin monotherapy	47 (44.3)
Colistin in combination with other antibiotics	29 (27.3)
ATB therapy not including colistin	6 (5.7)
**Outcome**	
Discharged from hospital	15 (14.2)
Fatal outcome in ICU	91 (85.8)

* Some patients had more than one comorbidity; ATB—antibiotic; BSI—bloodstream infection; COPD—chronic obstructive pulmonary disease; ICU—intensive care unit.

**Table 2:** The antibiotic resistance pattern of the *A. baumannii* isolates in healthcare-associated bloodstream infections in the COVID-19 intensive care unit at the Dubrava University Hospital during the second wave of COVID-19 pandemic (1 October 2020–28 February 2021) (N = 112).

Antibiotic	Proportion of Resistant Isolates N (%)
Imipenem	112 (100.0)
Meropenem	112 (100.0)
Ciprofloxacin	112 (100.0)
Gentamicin	111 (99.1)
Amikacin	100 (89.3)
Trimethoprim/sulfamethoxazole	43 (38.4)
Ampicillin/sulbactam	11 (9.8)
Colistin *	4 (3.8)

* The number of isolates tested for colistin varies (N = 105).

## Data Availability

The data presented in this study are available upon request from the corresponding author. The data are not publicly available due to privacy and ethical restrictions.
